# Information Dynamics in Urban Crime

**DOI:** 10.3390/e20110874

**Published:** 2018-11-14

**Authors:** Miguel Melgarejo, Nelson Obregon

**Affiliations:** 1 Laboratory for Automation and Computational Intelligence, Universidad Distrital Francisco José de Caldas, Bogotá 110311, Colombia; 2 Institute for Geophysics, Pontifical Xaverian University, Bogotá 110311, Colombia

**Keywords:** information, multifractal analysis, dynamics, chaos, crime

## Abstract

Information production in both space and time has been highlighted as one of the elements that shapes the footprint of complexity in natural and socio-technical systems. However, information production in urban crime has barely been studied. This work copes with this problem by using multifractal analysis to characterize the spatial information scaling in urban crime reports and nonlinear processing tools to study the temporal behavior of this scaling. Our results suggest that information scaling in urban crime exhibits dynamics that evolve in low-dimensional chaotic attractors, and this can be observed in several spatio-temporal scales, although some of them are more favorable than others. This evidence has practical implications in terms of defining the characteristic scales to approach urban crime from available data and supporting theoretical perspectives about the complexity of urban crime.

## 1. Introduction

Crime is not what it looks like. Despite its apparent random configuration over time, space, and society, crime forms patterns [1]. According to crime pattern theory, a pattern is a plausible interconnection between objects, rules, or processes that can be observed from practical experience or inferred from a theoretical basis [2]. Although this formulation is conceptually sound, crime patterns might not be evident, such that going deeper into the evidence is necessary to detect them. One important feature of crime patterns is their inherently dynamic nature [3] which makes their detection a challenge. The macro-level dynamics of socio-technical systems is counter-intuitive because of the nonlinear entanglement of diverse elements in the system [4]. Hence, detecting crime patterns should consider what complexity science can offer [5].

The emergence of patterns in urban crime is related to the complexity of cities [6,7]. Moreover, from environmental criminology, these patterns arise because crime is a decisional process motivated by the presence of opportunities in an urban backcloth [8] and supported by the bounded rationality of offenders [9]. Thus, the relation between these two perspectives motivates the exploration of ways to find some common ground [4].

With the development of geographical information systems, crime patterning has become in an intense research area [10]. Patterns are searched mainly in the spatio-temporal domain of crime by using statistical tools on reported crime data. Two problems have been observed while following this approach: the modifiable areal unit problem (MAUP) [11] and the crime aggregation problem (CAP) [12]. The former arises when geographical data such as crime counting are aggregated in spatial units. The size, shape, and orientation of these units produce a bias in statistical results. The latter appears when data of similar crimes are aggregated, which blurs the spatial distribution of crime occurrences that would hinder the detection of patterns.

Spatio-temporal patterns of crime have been studied from several perspectives to deal with the inherent uncertainty of this phenomenon. From probability and stochastic processes theories, crime patterning deals with fixing a probability distribution model over criminal data. Methods such as kernel density estimation (KDE) and self-exciting point processes (SPPs) among others have been considered in this context [13]. However, these tools usually base their assumptions on linearity, independence, stationarity, and ergodicity, which are not necessarily properties of criminal phenomena [14]. Additionally, statistical biases appear when using these approaches because of MAUP and CAP problems.

Stochastic approaches are sometimes used on the basis of a supposed similarity between crime and other phenomena. For example, SPP proposed to study crime more like a metaphor of seismic processes rather than a consequence of the nature of crime [15]. In another example, the movements of criminal offenders are modeled as random walks, which is far from the purposeful way people move through urban environments [16]. Other analyses of criminal dynamics take for granted the Poisson distribution (or other simple models) [17] of criminal attacks just because of the simplicity or popularity of this distribution, without first establishing its necessity. Models that rely on this assumption fail to account explicitly for the intricacies of urban crime dynamics, representing instead only some smooth attributes.

The analysis and detection of criminal patterns by means of clustering techniques have also been considered [18]. These studies focus on detecting groups of criminal events by looking for a particular kind of prototype (i.e., geometric shapes such as circles or ellipses). However, the problem of detecting criminal patterns in this way is the possible unmatching between the structure of crime data and clustering prototypes. In addition, fixing the number of clusters is still an open question in the pattern recognition field. Most of the studies on clustering criminal events deal only with the spatial domain of the phenomenon while ignoring the dynamic dimension of crime [19].

Recently, some artificial intelligence (AI) models for predicting crime patterns have been developed, with interesting results [20]. These approaches take advantage of methodological frameworks such as big data analysis and deep learning to establish nonlinear correlations between a large number of variables (i.e., economic, social, technological, climatological, etc.) and criminal patterns. However, these models lack explanatory power because of their complex correlative structure. Thus, minimal insight about the geometric structure of criminal patterns can be obtained from them.

Supported on crime pattern theory, risk terrain modeling (RTM) has been proposed as a promissory method to deal with the problem of detecting crime patterns [3,7,21]. In contrast to KDE and SPP, which rely on a frequentist interpretation of crime (i.e., counting), RTM uses Bayesian inference for crime patterning using prior information about the urban backcloth. This approach is more effective than KDE-based methods when sparse data about crime are available or crime rates are low [22]. As a method supported on parametric modeling, RTM relies on working suppositions and past experience to calibrate key parameters (e.g., cell size, bandwidth, etc.) [23]. However, the necessity of this calibration is not established from the information content of available data.

The crime patterning problem relies on the supposition that patterns are represented as probability distributions or geometric prototypes that can be easily parametrized. Thus, classical geometry is privileged when searching spatio-temporal patterns in crime data. If crime is the result of complex phenomena that emerge from the entanglement of multiple relations in urban systems, then searching for simple geometric patterns would be in contradiction with this hypothesis. Therefore, the crime patterning problem should be directed in a different way, in which the study of the geometric properties of urban crime becomes necessary.

Two new approaches that refocus the crime patterning problem were proposed in [14,24]. The former considers an entropy analysis of crime regions across several cities, revealing that crime concentrates dynamically. This result is interesting and provocative, but only one spatio-temporal scale for studying this dynamic is considered, which may limit the scope of the findings. The latter takes a look at the multifractal nature of crime dynamics by patterning long trends of criminal data for a particular city. The results of these studies are limited to one particular case showing that temporal crime dynamics resemble 1/f noise, and only some insights about the spatial properties of this phenomenon are given.

The geometry of urban crime is conditioned by its support, which is the city itself—not only in the physical domain (i.e., places, streets, architecture, etc.), but also in the social domain (i.e., people, economy, etc.) [25,26]. The properties of this geometry arise from the dynamical nature of cities. Therefore, in this work, the geometry and dynamics of urban crime are connected across space and time, defining a single category to study, which would require the identification of its characteristic scales.

This geometric perspective can take advantage of several tools that have barely been explored in the understanding of crime dynamics. Among them, one can find fractal/multifractal geometry and chaos theory. As will be discussed later, multifractal analysis gives insight into the apparent random geometry of urban crime in different spatio-temporal scales, while information production in these scales is studied on the basis of dynamical systems theory. Information is relevant in this work because it has been highlighted as one of the elements that shapes the footprint of complexity in natural and socio-technical systems [27]. In addition, information production as a dynamical process is a concept that would go beyond the traditional concepts of information theory [28].

Multifractal analysis (MFA) [29] and analysis of observed chaotic data (AOCD) [30] are combined in this work to characterize the information dynamics inside the geometry of urban crime. Urban crime is treated as a dynamic phenomenon, which is perceived through data obtained from police records and produces information over space and time. In addition, the proposed method can be used in practical terms to suggest characteristic scales for crime dynamics modeling purposes. Five cases (four cities in North America and one city in South America) are processed through this approach, which reveals that information production in the spatio-temporal dynamics of urban crime of these cities exhibit common patterns, such as low-dimensional chaotic attractors.

The application of MFA for studying complex phenomena has been discussed in the literature (e.g., [29,31,32]). Some works have investigated the fractal/multifractal nature of urban processes, such as in [6,33,34,35,36]. The concern for the fractality of crime appeared two decades ago in criminology [37], and recent efforts have tried to connect the complexity of urban systems to certain social phenomena, such as crime and violence, by looking at fractal/multifractal patterns [6,38].

This work follows this line of research to analyze the dynamic informational dimension of urban crime, which is connected at the root of the decisional and complex aspects of this phenomenon. The decisional process behind crime can be described by means of scripts that depict how agents rely on experience, environmental clues, and opportunities to refine their behavior [2,9]. Offenders use internal and surrounding information to produce crime information over time, space, and society. Thus, analyzing the dynamics of this production of information would be an unexplored perspective to detect spatio-temporal patterns in urban crime.

The rest of this paper is organized as follows: Section 2 summarizes some concepts about crime pattern theory, MFA, and AOCD. Section 3 presents the proposed approach to study the information dynamics in urban crime. This section also discuses some implications of the informational analysis of urban crime. Results obtained from the application of this approach over considered cities are presented in Section 4. Next, Section 5 discusses these results. Finally, we draw some conclusions in Section 6.

## 2. Preliminaries

### 2.1. Crime Pattern Theory and Related Perspectives

Crime pattern theory understands crime as a variety of complex phenomena that do not occur randomly in space, time, or society [39]. This theory focuses its attention on rules that can explain the non-randomness of crime dynamics observed from experience. Although patterns can be obvious in some situations, delving deeper into the context is necessary to detect them in other cases. Crime patterns are dynamic and appear at micro and macro levels with similar characteristics and rules [40]. Hence, crime exhibits scale-free behavior and it should be understood as a whole that covers not only the decisional processes of actors but also the urban backcloth that surrounds them.

Routines are the mechanisms behind the formation of crime patterns. Actors perform daily routines that interact over the urban support, generating crime opportunities [41]. Sometimes, triggering events appear associated to those opportunities that lead to the production of offenses. Although an offense is motivated by a triggering event, it demands a decision to carry it out. This decision can be depicted as a template that illustrates how the performer follows a learning process using cumulative experience and the interaction with a social network.

Two perspectives that complement crime pattern theory have been proposed in order to explain some details about the decisional process behind the execution of a crime event. The rational choice perspective suggests that crime is a decision-making process under uncertainty [9]. Criminal behavior is purposive and supported by a bounded rationality that evaluates the risk and benefits of offending [42,43]. Offenders try to predict the possible outcomes of their choices, which is prone to errors due to several constraints. As the process evolves by learning, the chances of success improves. Disruptions may sometimes appear, providing new experiences that help to refine this process. The whole picture can be depicted as a script, giving the idea of a heuristic that maximizes the benefits of the offender.

The routine activity approach explains how crime rates emerge [8]. This approach considers two levels of analysis: on a micro level, it states that an ordinary crime emerges as the convergence of a likely offender and a suitable target given the absence of inhibitors. On a macro level, it suggests that certain features of the socio-technical system that surrounds offenders and targets increase the likelihood of such convergences [44]. In addition, the routine activity approach states that a crime is a rare event that comes from routine events. Thus, offenders and targets exhibit dynamic behaviors that interact in fine temporal scales. Offenders often operate in association, which makes this dynamics complex. These considerations have linked crime to some life processes, which has motivated an appeal to life sciences to study it [45].

### 2.2. Multifractal Analysis

#### 2.2.1. Multifractal Spectrum

Consider an object that is covered with counting boxes of longitude *L*. The local density $P_{i}\left(L\right)$ of the object is a mass function of the *i*-th counting box:

(1) $$P_{i}\left(L\right)=\frac{M_{i}\left(L\right)}{M_{T}},$$

where $M_{i}\left(L\right)$ is the number of pixels that contribute to the mass in the box and $M_{T}$ is the total mass of the object.

In heterogeneous objects, $P_{i}\left(L\right)$ can vary as [46]:

(2) $$P_{i}\left(L\right)∼L^{\alpha_{i}},$$

where $\alpha_{i}$ is the Lipschitz–Holder exponent that characterizes the scaling of the *i*-th region or spatial location. These exponents show the local behavior of $P_{i}\left(L\right)$ around the center of a counting box with longitude *L*. Most of the time, similar values for $\alpha_{i}$ are found in different regions of the object.

The number of boxes N(α) where the mass function has exponents between α y α+dα scales as:

(3) $$M\left(\alpha \right)∼L^{-f(\alpha )},$$

where f(α) can be defined as the fractal dimension of the set of boxes whose exponent is α.

Multifractal measures appear as scalings of the *q*-th moments of the density function $P_{i}\left(L\right)$ [29]:

(4) $$\sum_{i=1}^{M(L)}P_{i}^{q}\left(L\right)=L^{\left(q-1\right)}D_{q},$$

where $D_{q}$ are the generalized fractal dimensions. According to [31]:

(5) $$D_{q}=\frac{1}{q-1}\lim_{L\to 0}\frac{\log\sum_{i=1}^{M(L)}P_{i}^{q}\left(L\right)}{\log L}.$$

The exponent in Equation (4) is called the mass exponent of the *q*-th moment of order τ(q):

(6) $$\tau \left(q\right)=\left(q-1\right)D_{q},$$

with

(7) $$\alpha \left(q\right)=\frac{d\tau (q)}{dq}.$$

**Figure 1:**
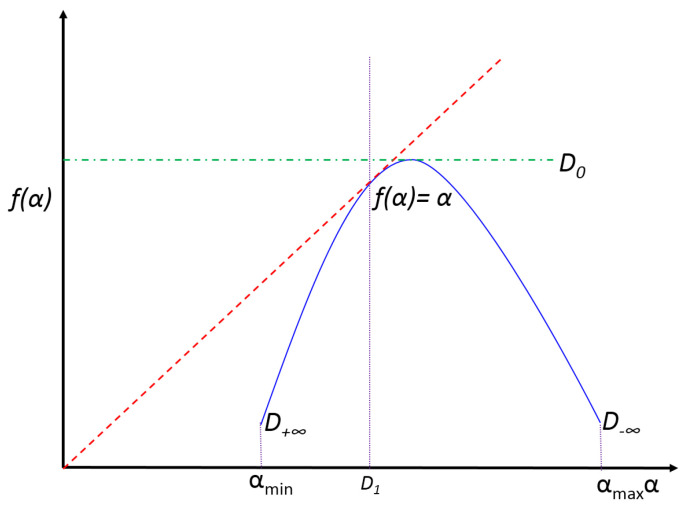
The graph f(α) vs. α or multifractal spectrum. The spectrum is a concave function whose maximum coincides with the fractal dimension of the object $D_{0}$. Its intersection with the identity line is the information dimension $D_{1}$. The asymmetry of the spectrum is related to the abundance of high or low masses in the object. The wider the spectrum, the more multifractal the object is.

The curve f(α) vs. α is called the *multifractal spectrum*, which is a convex function with a maximum $D_{0}$ in q=0 (fractal dimension), as shown in Figure 1. For q=1, $f\left(\alpha \right)=\alpha =D_{1}$, which is called the information dimension. $D_{0}$ and $D_{1}$ characterize the occupation of the support and the scaling of information production, respectively. The difference between $\alpha_{min}$ and $\alpha_{max}$ reveals the width of the set of local scales or how strong the mutlifractality of the object is. In addition, $D_{-\infty }-D_{+\infty }>0$ is an index of the spectral symmetry, which is related to the abundance of regions with small masses [47].

Practically speaking, the multifractal spectrum cannot be computed in the infinity, so its estimation is limited to the set of local scales that can be expressed as powers of *L*. This also restricts the range of moments *q* that can be used. Therefore, the multifractal spectrum is computed from:

(8) $$\mu_{i}\left(q,L\right)=\frac{P_{i}^{q}\left(L\right)}{\sum_{i=1}^{M(L)}P_{i}^{q}\left(L\right)},$$

where $P_{i}\left(L\right)$ is a fraction of the amount of pixels in each box of longitude *L*.

Thus, the computation of f(q) and α(q) goes as follows [46]:

(9) $$f\left(q\right)=\lim_{L\to 0}\frac{H(L)}{\log L}=\lim_{L\to 0}\frac{\sum_{i=1}^{M(L)}\mu_{i}\left(q,L\right)\log\mu_{i}\left(q,L\right)}{\log L},$$

(10) $$\alpha \left(q\right)=\lim_{L\to 0}\frac{W(L)}{\log L}=\lim_{L\to 0}\frac{\sum_{i=1}^{M(L)}\mu_{i}\left(q,L\right)\log P_{i}\left(L\right)}{\log L}.$$

Because these expressions cannot be evaluated directly, it is necessary to estimate f(q) and α(q) for each *q* as the slope of the linear regression of numerators in Equations (9) and (10) versus logL over the range considered for *L*. The goodness of the regression should be evaluated to determine which scales define the multifractal behavior of the object. Moreover, it is appropriate to observe other properties of the spectrum, such as concavity and tangentiality to the identity line, as a way of evaluating its consistency. In practical terms, the concavity of the spectrum depends on the range of *L*, particularly on its minimum $L_{min}$, which corresponds to the smallest scale in which the object exhibits multifractal behavior given the available data.

#### 2.2.2. Information Dimension $D_{1}$

Note the singularity 1/(q−1) in the evaluation of $D_{q}$ in Equation (5). Therefore, special attention is needed in this computation when q=1. In the limit q→1, it can be shown that [29]:

(11) $$\log\left(\sum_{i}P_{i}^{q}\right)\to \log\left\{1+\left(q-1\right)\sum_{i}P_{i}\log P_{i}\right\}≃\left(q-1\right)\sum_{i}P_{i}\log P_{i}.$$

In this way, Equation (5) can be expressed as follows:

(12) $$D_{1}=\lim_{L\to 0}\frac{\sum_{i}P_{i}\log P_{i}}{\log L}.$$

Multiplying by −logL on both sides of Equation (12):

(13) $$-D_{1}\log L=-\sum_{i}P_{i}\log P_{i}.$$

Considering a probabilistic interpretation of the mass densities $P_{i}\left(L\right)$, the right-hand side of Equation (13) is the *informational entropy E* of P(L):

(14) $$E\left(L\right)=-D_{1}\log L.$$

*E* is given straight forward by $D_{1}$ and scales logarithmically with *L*.

### 2.3. Analysis of Observed Chaotic Data

#### 2.3.1. Taken’s Theorem

Chaos is a phenomenon that appears in some signals. Its footprint is characterized by several attributes, such as complex dynamical traces in time, broadband power density spectra, nonperiodic motion, and exponential sensitivity to reduced perturbations in the orbit of the phase plane, among others [48]. Although chaos is irregular in time and is slightly predictable, it exhibits structure in the phase space [30]. Most studies of chaos center their analysis on nonlinear dynamic systems whose governing equations are well established [49]. However, some studies have attempted to infer the presence of chaos in a signal only by means of available data. Chaos detection is useful when there is no knowledge about the structure of the system that produced the signal [50].

Taken’s embedding theorem [30,51] gives us a way to represent an equivalent phase space for the dynamics that produced the observed signal s(n). This theorem guarantees the reconstruction of the geometric structure of the dynamics that shapes the signal. In this sense, a dynamical system can be represented as follows:

(15) x(n)→F(x(n))=x(n+1),

where x(t) is a multidimensional phase space. If a scalar quantity h(∘) of some vector function g(x(n) is known, then the geometry of the dynamics can be unfolded from the mapping h(g(x(n)) as a new vector space. Each vector consists of elements in which h(∘) is applied to powers of g(x(n)), as denoted:

(16) $$y\left(n\right)=[h\left(x\left(n\right)\right),h\left(g^{T_{1}}\left(x\left(n\right)\right)\right),h\left(g^{T_{2}}\left(x\left(n\right)\right)\right),\cdots ,h\left(g^{T_{d-1}}\left(x\left(n\right)\right)\right)].$$

y(n) defines a motion in a d-dimensional Euclidean space. Some properties of chaos are reproduced in the new space as y(n)→y(n+1) evolves in time following the unknown dynamics given by Equation (15). Since x(n)→x(n+1) is deterministic, the substituting dynamics y(n)→y(n+1) will also be.

Considering a general scalar function h(∘) and a general function g(x) consisting of some initial vector and its time-delayed versions, y(n) just contains time lags of the observed signal:

(17) y(n)=[s(n),s(n+T),s(n+2T),⋯,s(n+(d−1)T)],

where s(n)=h(x(n)) and $T_{k}=kT$.

Regarding Equation (17), it is necessary to identify two parameters: the time delay *T* between delayed versions of s(n) and the number of these versions, which is called the global dimension $d_{E}$ of the phase space that contains the underlying dynamics of the system.

#### 2.3.2. Average Mutual Information

In the case of *T*, its identification should guarantee that this delay is large enough so that s(n) and s(n+T) are slightly independent but not too large that these signals are entirely statistically independent [30]. Thus, *T* can be established in terms of the information among measurements, which is expressed as the *average mutual information* (AMI) I(T) between delayed versions s(n) and s(n+T), given by:

(18) $$I\left(T\right)=\sum_{s(n),s(n+T)}F\left(s\left(n\right),s\left(n+T\right)\right)\log_{2}\left[\frac{F(s(n),s(n+T))}{F(s(n))F(s(n+T))}\right],$$

where F(s(n),s(n+T)) is the joint probability density between signals s(n) and s(n+T), whereas F(s(n)) and F(s(n+T)) are individual probability densities.

Thereby, Equation (18) can be understood as a nonlinear correlation function, which helps to determine when s(n) and s(n+T) are sufficiently independent to work as coordinates in a time delay vector y(n). A plausible size of the time lag *T* is obtained by exploring around the first minimum of the nonlinear auto-correlation function I(T). The time delay *T* can be computed by other means, such as the *correlation integral*, to validate the results obtained from AMI. However, linear correlation is not recommended as a confirmation method for the time delay because it can be fooled by nonlinear dynamics [52].

#### 2.3.3. False Nearest Neighbors

With the candidate delay *T* that was suggested by the AMI computation, a phase space reconstruction is carried out given a dimension *d* in Equation (17). An examination of the nearest neighbors in phase space of the vector y(n) follows as:

(19) $$y^{w}\left(n\right)=\left[s^{w}\left(n\right),s^{w}\left(n+T\right),s^{w}\left(n+2T\right),\cdots ,s^{w}\left(n+\left(d-1\right)T\right)\right].$$

Two possible situations can occur regarding the proximity of y(n) and $y^{w}\left(n\right)$ [53]. In the former, $y^{w}\left(n\right)$ comes to the neighborhood of y(n) through dynamical origins, which implies movements along similar orbits around the attractor. In this case, each point of the phase space is surrounded by numerous neighbors, and the state space is populated if enough data are collected. In the latter, $y^{w}\left(n\right)$ is a false neighbor of y(n), which means that it has arrived to the neighborhood of y(n) as a result of the projection from a higher dimension because the current dimension *d* does not unfold the attractor. By moving up to the next dimension d+1, the false neighbors will be outside the neighborhood of y(n).

The proximity between y(n) and $y^{w}\left(n\right)$ can be estimated by means of the Euclidean distance $U_{d}\left(n\right)$:

(20) $$\begin{array}{cc} U_{d}^{2}\left(n\right)= & {\left[s\left(n\right)-s^{w}\left(n\right)\right]}^{2}+{\left[s\left(n+T\right)-s^{w}\left(n+T\right)\right]}^{2}+{\left[s\left(n+2T\right)-s^{w}\left(n+2T\right)\right]}^{2}+\cdots \\ & +{\left[s\left(n+\left(d-1\right)T\right)-s^{w}\left(n+\left(d-1\right)T\right)\right]}^{2}. \end{array}$$

When the dimension is increased to d+1, the distance changes according to:

(21) $$U_{d+1}^{2}\left(n\right)=U_{d}^{2}\left(n\right)++{\left[s\left(n+\left(d\right)T\right)-s^{w}\left(n+\left(d\right)T\right)\right]}^{2}.$$

If $U_{d+1}\left(n\right)$ becomes much larger than $U_{d}\left(n\right)$, then it may be given by the effect of some neighbors that appear from the projection of a higher-dimensional attractor. The following ratio be can used to decide if this increment is significant, which reveals the presence of false neighbors:

(22) $$\frac{|s\left(n+Td\right)-s^{w}\left(n+Td\right)|}{U_{d}\left(n\right)}>U_{N}.$$

According to practical observations, the number of false neighbors remains constant when $10\leq U_{N}\leq 50$. The dimension *d* in which false neighbors become minimum is selected as the embedding dimension of the dynamics y(n). Empirical evidence shows that false neighbors drop to zero in deterministic low-dimensional motion. In contrast, residual false neighbors result from truly stochastic or high-dimensional chaotic data [30,50]. If deterministic dynamics is detected, then it is interesting to estimate the largest Lyapunov exponent (LLE) of the time series s(n) because positive LLEs appear generally in chaotic motion. Several algorithms have been proposed to carry out this estimation [52,54,55,56], but most of these methods are sensitive to the amount of available samples, which might restrict their application.

## 3. Materials and Methods

### 3.1. Criminal Reports

A criminal complaint is defined as a tuple z={xyt}, where x∈R, y∈R are the spatial attributes and t∈N is the temporal attribute of the complaint. These attributes will be noted as $z^{x}$, $z^{y}$, and $z^{t}$, and they configure a perception about where and when the criminal event happened. In some cases, all attributes can be established without uncertainty. However, in others only inaccurate information about the event is available [11].

A criminal report *R* is the set of all criminal complaints between the time interval $\Delta t=t_{fin}-t_{ini}$:

(23) $$\begin{array}{cc} R= & \{z_{1},z_{2},\cdots ,z_{N}\},\\ t_{fin}= & \max\left(z_{j}^{t}\right),t_{ini}=\min\left(z_{j}^{t}\right). \end{array}$$

A criminal subreport in a time window Δv≤Δt is a subset $\hat{R}$ of *R*:

(24) $$\begin{array}{cc} \hat{R}= & \{z_{1},z_{2},\cdots ,z_{Q}\},\\ t_{fin}\geq & \max\left(z_{j}^{t}\right),t_{ini}\leq \min\left(z_{j}^{t}\right), \end{array}$$

where *Q* is the amount of registered complaints in the interval Δv.

Two criminal subreports ${\hat{R}}_{1}$ y ${\hat{R}}_{2}$ are disjunctive if:

(25) $${\hat{R}}_{1}⋂{\hat{R}}_{2}=\emptyset .$$

A criminal report is a collection of disjunctive criminal subreports:

(26) $$R=\overset{M-1}{\underset{n=0}{⋃}}\hat{R}\left(n\right),$$

where *M* is the total number of criminal subreports.

**Figure 2:**
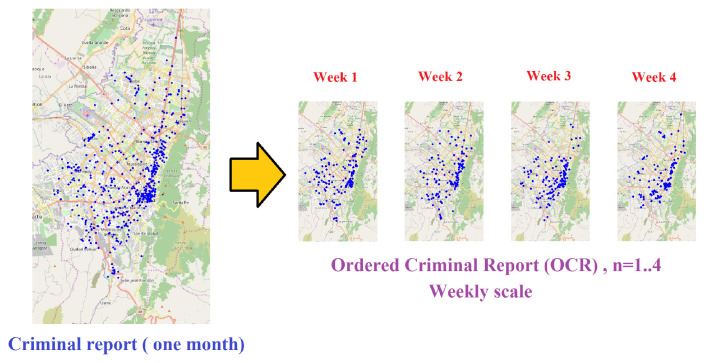
An illustrative example of an ordered criminal report (OCR) that covers one month of crime complaints. In this example, events corresponding to one month of criminal activity have been aggregated and then splitted into four disjunctive weekly subreports.

An ordered criminal report (OCR) is a criminal report with:

(27) $$\max\left[z_{j}^{t}\left(n\right)\right]<\min\left[z_{i}^{t}\left(n+1\right)\right],$$

which guarantees that consecutive subreports $\hat{R}\left(n\right)$ and $\hat{R}\left(n+1\right)$ are disjunctive in an OCR. An illustrative example of an OCR is depicted in Figure 2, in which a criminal report of one month has been decomposed in four disjunctive sub-reports, each one covering one week. Criminal complaints are geotagged over a representation of the city support, which is given primarily as the street network. Each geotag includes the spatial and temporal attributes of the complaint given a coordinate system that is well-suited for the city. The temporal attribute of complaints allows the subreports to be ordered in the OCR. Most of real-world reports are plagued with uncertainty because of not only the deficiency in precision of times and locations of complaints, but also the level of under-report [11,12].

### 3.2. MF Time Series

The application of multifractal analysis to each subreport $\hat{R}\left(n\right)$ of an OCR *R* produces a sequence of multifractal spectra f(α(n)) (this conceptualization is similar to that proposed in [57]). From the definition of an OCR, the subreports $\hat{R}\left(n\right)$ are disjunctive, guaranteeing that the generation of f(α(n)) does not share criminal complaints between the moments *n* and n+1. Therefore, f(α(n)) can be interpreted as a dynamic multifractal spectrum. That is, a multifractal spectrum whose parameters change over time, each of them giving rise to a time series.

**Definition.**  *An* MF time series *S(n) is the sequence of values produced by some statistic obtained from a dynamic multifractal spectrum with n=0,⋯,M−1.*

To evaluate the quality in the generation of f(α(n)), given a minimum scale (minimum box longitude) $L_{min}$, in terms of its MF time series, we introduce the concavity index CI(n):

(28) $$CI\left(n\right)=\left\{\begin{array}{cc} 1 & \mathrm{if}\;D_{1}\left(n\right)\leq D_{0}\left(n\right)\;\mathrm{and}\;D_{+\infty }\left(n\right)\leq D_{1}\left(n\right)\;\mathrm{and}\;D_{-\infty }\left(n\right)<D_{0}\left(n\right),\\ 0 & \mathrm{otherwise}. \end{array}\right.$$

The cumulative concavity index CCI for f(α(n)) from CI(n) is computed as follows:

(29) $$CCI=\frac{1}{T}\sum_{n=0}^{M-1}CI\left(n\right).$$

A concavity test is proposed to accept or reject the generation of f(α(n)). The MF time series are accepted when CCI>0.95, but the desirable situation regarding the generation of these series should be CCI=1.0. When the ideal condition is not met, then degenerated spectra f(α(n)) should be identified and, if possible, corrected.

### 3.3. MF-A2-OCD Method

**Figure 3:**
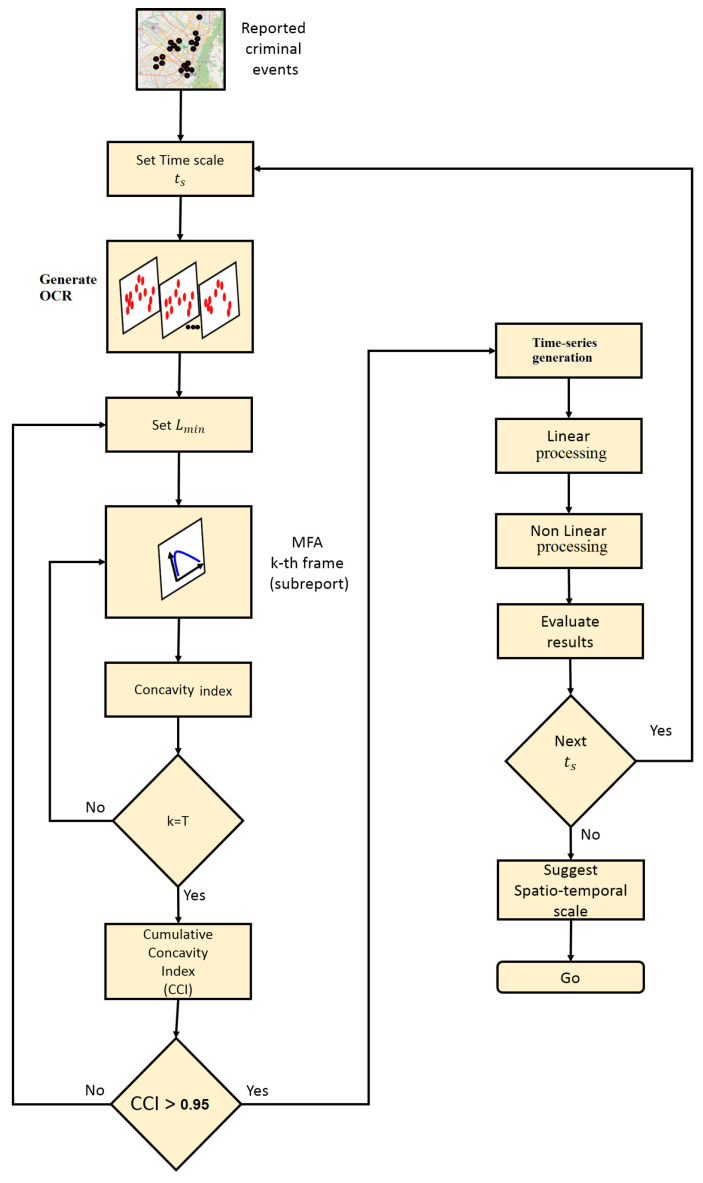
Proposed approach that combines multifractal analysis (MFA) and analysis of observed chaotic data (AOCD) (MF-A2-OCD) to study information dynamics in urban crime reports. The method focuses on detecting spatio-temporal scales where information production exists in crime reports given that multifractal behavior appears to be consistent.

This method is proposed to study the temporal structure of MF time series by means of the analysis of observed chaotic data (AOCD). The MF-A2-OCD method is depicted in the flow diagram of Figure 3, which is described as follows:

- *Generate the OCR*: Given the record of urban crime complaints in a time window Δt, a temporary scale $t_{s}$ is defined for the construction of the OCR. Depending on the scale chosen, the report will contain *T* disjunctive subreports $\hat{R}\left(n\right)$. The index *n* reveals the order in occurrence of the subreports over the OCR and will refer to the day, week, or month of the subreport $\hat{R}$ within the OCR, depending on the selected scale.
- *Multifractal analysis and concavity test*: Given a minimum spatial scale $L_{min}$, multifractal analysis is executed for each of the subreports $\hat{R}\left(n\right)$. The multifractal analysis is standardized considering for all the cases the same sizing of the support given by the maximum and minimum of the spatial coordinates of all complaints in the OCR. The concavity index of each spectrum f(α(n)) is obtained according to Equation (28), until completing the length of the OCR *M*. Then, the CCI is obtained and the concavity test is verified, and if negative a new $L_{min}$ is chosen and the MF analysis is executed again. In practical terms it is desirable to start with a small $L_{min}$ and increase it until the test becomes positive, keeping in mind the possible degeneration of some multifractal spectra that should be corrected.
- *Synthesis of MF time series*: The signals $D_{0}\left(n\right)$, $D_{1}\left(n\right)$, $D_{-\infty }\left(n\right)$, $D_{+\infty }\left(n\right)$,$\alpha_{min}\left(n\right)$ and $\alpha_{max}\left(n\right)$ are constructed from the accepted dynamic multifractal spectrum f(α(n)). For those spectra whose concavity index is at zero, the value of MF time series can be recalculated using a larger $L_{min}$. However, there is no guarantee of achieving the concavity of the spectrum despite this increase, because it will depend on whether there are enough complaints in the subreports that configure objects with at least monofractal behavior. Other mechanisms can be used to fix these values, such as filling methods that preserve local statistics of the signal around problematic values [58].
- *Linear processing*: Linear statistics are computed over produced MF time series, such as: autocorrelation function, power spectrum, mean estimation, variance estimation, and coefficient of variation, among others. It is recommended to complement this analysis with the calculation of the signal histogram. The autocorrelation and the power spectrum make it possible to determine if there are any periodic behaviors within the signal detectable in a linear sense. These two statistics have a special link through the Wiener–Khinchin [59] theorem. The other statistics are calculated in order to have an appreciation of the overall behavior of the signal [30,60].
- *Nonlinear processing*: In this stage, a battery of nonlinear statistics is applied to explore the structure of the time series to reveal details of its behavior that escape the linear analysis [30,50]. Some of the statistics that can be considered here are: average mutual information, dimension of the embedded phase space, and estimation of the maximum Lyapunov exponent, among others, which are based on the theory of dynamic systems, particularly nonlinear and chaotic systems [49,55,56]. Other approaches related to the detection of chaos in time series may consulted in [61]. This analysis can be complemented from a statistical perspective with an indicator of self-similarity and predictability, such as the Hurst exponent [14,29,37].
- *Characteristic scales*: In addition to the results produced from previous stages, spatio-temporal scales are suggested to approximate the understanding of the phenomenon. The CCI reveals the minimum scale over which the temporal consistency in the mutifracatal properties of the phenomenon in space can be judged, manifesting itself as a sequence of coherent multifractal spectra, on which an attempt has been made to minimize the effect of degeneration. Results from linear processing may reveal the conservation of a spatial multifractal characteristic that can be predictable at a certain time scale. Meanwhile, the results from nonlinear processing indicate to what extent this characteristic may be chaotic, which would limit the prediction horizons in a certain time scale.

### 3.4. Information Scaling in Crime Reports

Informational entropy is a measure of the average information content of a set’s density (i.e., probability) distribution. The occurrence of rare events increases this content, whereas common events produce just a small increase of it. Therefore, according to the routine activity approach, if crime offenses are rare events that emerge from the interaction of routine events, densities may produce a significant average content of information. On the contrary, if crime events populate in certain locations, corresponding densities may reduce the informational entropy of the distribution.

The maximum informational entropy is achieved when a probability distribution is uninformative. Typically, this situation appears when there is no prior knowledge about the phenomenon so that the best distribution that supports any decision is the one with the highest informational entropy. For example, if no constraints are given, the uniform distribution is the best choice. In the framework of crime pattern theory, crime does not distribute uniformly, so the crime decisional processes would modulate crime distributions, reducing their informational entropy. Therefore, an observer would note that crime distributions over space or time would become more informative as the learning processes of offenders improve. However, there are other elements that may contribute to shape the distribution, since crime is connected to the urban backcloth.

**Figure 4:**
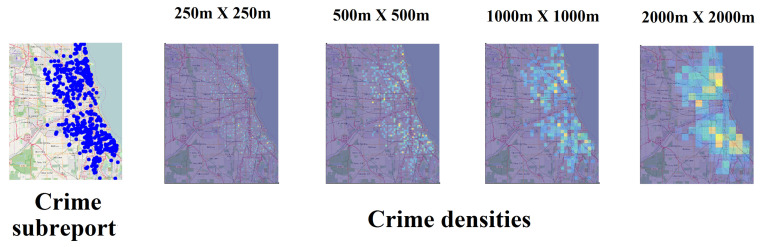
Example of crime densities in four spatial scales when computing the multifractal spectrum of a crime subreport. Spatial patterns of crime densities change with scale, although some characteristics are preserved.

The quantification of crime densities $P_{i}$ requires the definition of a scale *L*. This makes the same set of criminal events to configure different spatial patterns depending on *L*, but some similarity can be noted between several scales, as shown in Figure 4. As the scale becomes larger, more crime events are aggregated in the areal units, which induces one to think about the presence of patterns that are not evident in the smallest scales. This dependence of densities in relation to the areal unit impacts any statistical characterization, including the informational entropy.

**Figure 5:**
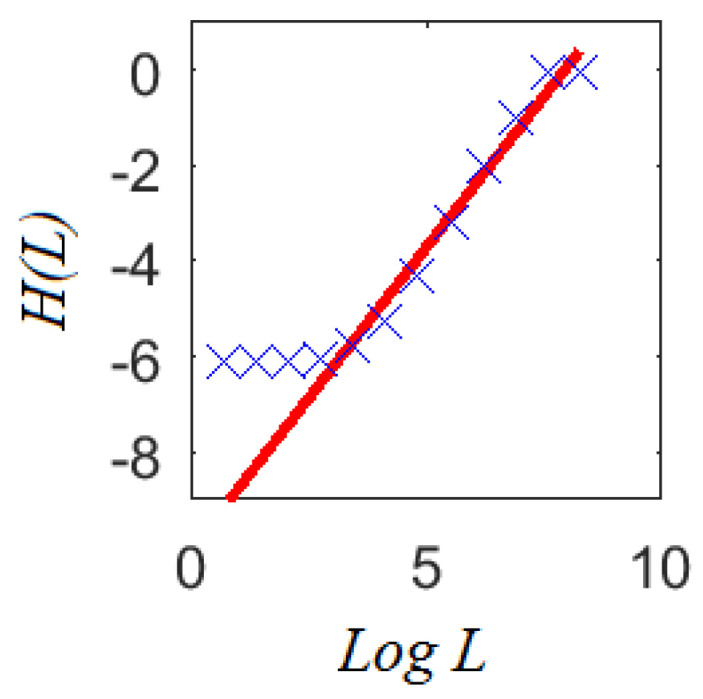
An example of how the information dimension $D_{1}$ is obtained from multifractal analysis of the crime subreport in Figure 4. Linear scaling of the informational entropy is observed for a limited set of scales which corresponds to where multifractal behavior appears. Multifractality is not observed in all scales since there is only a finite number of points in the crime subreport.

The theoretical result provided by multifractal analysis in Equations (11)–(13) shows how the informational entropy scales with the logarithm of the areal unit size *L* (i.e., a L(m)×L(m) box). In practical terms, this scaling can be estimated from the curve H(L) vs. LogL in Equation (9) applied over crime masses at different scales with moment q=1.0, as shown in Figure 5. Note that H(L) at the smallest and largest scales does not exhibit significant changes. Notorious changes in H(L) are observed at middle scales, following a linear dependence with LogL.

The slope of the fitted linear regression corresponds to the informational dimension $D_{1}$. This is interesting because scales where the informational entropy grows linearly with LogL correspond to those where multifractal behavior can be appreciated. Although crime densities look different from one scale to another, there is a set of scales where their average informational content scales linearly as $D_{1}\times LogL$. This feature gives strong support to the idea that crime patterns at different spatial scales share common properties (as suggested by crime pattern theory), at least the rate $D_{1}$ as the informational entropy increases.

According to Equation (13), information production will be present for any scale in a multifractal object. However, this is not the case for a crime report since this property only appears over a limited set of scales, as can be seen in Figure 5. In practical terms, detecting this set sheds light on the spatial scales where information exists to perform any complementary statistical analysis. Therefore, the analysis of information scaling would help to deal with the problem of selecting adequate areal units for aggregation purposes, for example when sparse spatial data is available [62]. Moreover, the identification of characteristic scales would suggest the smallest one where information scaling starts given the available data. This may suggest if patterns will be identified when using a fine segmentation of crime events.

### 3.5. Information Patterns in Ordered Crime Reports

Informational entropy is related to information scaling in a multifractal object. The MF-A2-OCD method looks to obtain a consistent multifractal behavior from an OCR in order to guarantee the integrity of information scaling over the sequence of disjunctive crime subreports. Thus, the $D_{1}\left(n\right)$ series would capture some insight of the spatio-temporal dynamics of reported crime at least in informational terms. The dynamic information content of crime can be approached as a signal processing problem so that temporal patterns might be detected or not by means of linear and nonlinear analyses.

The understanding of crime dynamics through informational patterns in time may help to detect correlations or seasonalities between disjunctive crime subreports. This approach would provide a general look at the memory structure of crime dynamics captured through an OCR considering a set of temporal scales. The absence of informational patterns may suggest that crime dynamics corresponds to a truly stochastic process. On the contrary, detecting these patterns would be a confirmation that crime dynamics exhibits a temporal structure that can be studied. Hence, the temporal non-randomness of the crime hypothesis at the core of crime pattern theory can be tested. In addition, this perspective can be used to contrast the information patterns in different temporal instances such as weekdays/weekends, night/day, or seasons, among other possibilities [63,64,65] in order to characterize the global memory of crime.

### 3.6. Research Data

Five cases of urban crime report in cities of America were considered, as follows: Los Angeles (USA), Chicago (USA), Philadelphia (USA), San Francisco (USA), and Bogota (Colombia). The choice of these cases was mainly due to the availability of open criminal databases. In these five cities, criminal reports cover 1237 days (i.e., 176 weeks or 44 months), extending from January 2012 to May 2015. The length of the reports was standardized with respect to the Bogota case, which is the shortest. The minimum time scale of analysis was daily, given that not all reports recorded information on an hourly scale.

Records focus solely on property crimes, which could involve violence but not weapons [10,66]. In particular, the records considered in the city of Los Angeles, Chicago, and Philadelphia covered robberies (i.e., theft), assaults (i.e., robbery), and raids (i.e., burglary). In San Francisco, the complaints focused on raids, while in Bogota the reports focused on thefts. The aggregation given in the first three cities was carried out only from a practical point of view to avoid daily or weekly empty subreports, which is a typical practice in view of the deficiency of recorded complaints. The convenience of aggregating between types of crimes is an open topic within space criminology because similar types of crimes do not necessarily generate similar spatial patterns [12,67]. In this sense, the cases considered in this investigation cover two situations in relation to the crime aggregation problem.

Table 1 presents a collection of relevant data of cities and their respective criminal reports. It has been suggested that criminal activity is positively correlated to the area and the population size of a city [6,68], and also to socio-economic aspects that can be expressed as indicators of well-being and inequality [69]. Note that these cities cover an interesting range of areas and population sizes, whereas the criminal reports span about one order of magnitude in size and average daily complaints. These cities are characterized by the convergence of a large amount of economic, social, and technical activities. In addition, observe that in terms of the welfare indicator (GDP), which involves aspects such as health, education, economic benefits, and civic environment, the cities in the United States exhibit similar levels, while the Bogota case is notably lower.

## 4. Results

### 4.1. Multifractal Analysis of Crime Subreports

**Figure 6:**
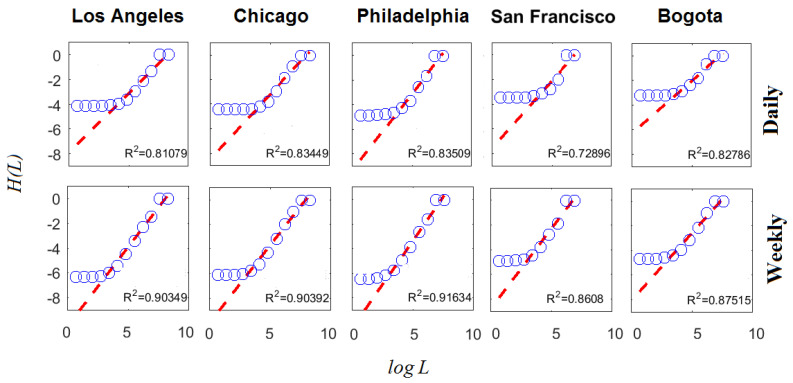
$D_{1}$ estimation for subreports with the largest number of criminal complaints. Note that information production measured through the informational entropy scaled linearly with LogL only in a finite set of scales. The determination coefficient $R^{2}$ validates the goodness of the linear regression that was used to estimate the information dimension $D_{1}$ (i.e., slope of the linear regression).

**Figure 7:**
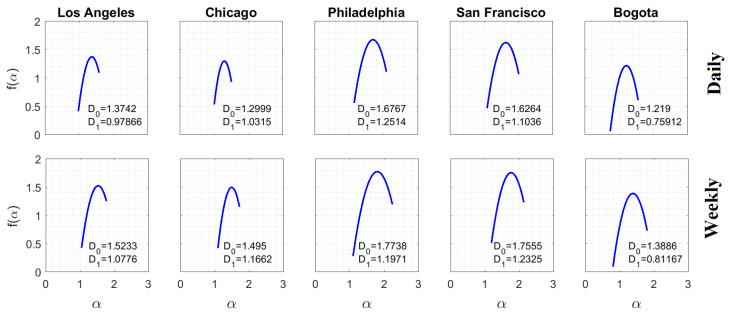
Multifractal spectra of subreports with the largest number of criminal complaints. The spectra show that these crime reports exhibited multifractal behavior over the scales in which the informational entropy scaled linearly with LogL. Both the fractal dimension $D_{0}$ and the information dimension $D_{1}$ increased, in general, with the temporal scale.

Computation of Equation (9) (q=1.0 and $L=16\times 2^{l}$ m, l=0⋯10) for the daily and weekly subreports with the largest number of criminal complaints is presented in Figure 6, whereas the corresponding multifractal spectra are presented in Figure 7. This analysis is interesting because it helps to understand how information scales in space by the estimation of $D_{1}$, which corresponds to the slope of the linear regression H(L) vs. logL. The largest subreports were considered because their multifractal characteristic are the strongest.

Note that the quality of the adjustment of the linear regression on logL, expressed through the coefficient of determination $R^{2}$, increased as the time scale became coarser. This implies that information decrease remained constant over a greater number of spatial scales when the time scale increased. Thus, the informational self-similarity in space was limited to a smaller range of scales when the time scale became finer. In the daily case, this corresponded to the set of scales with L≥1000 m and in the weekly case it corresponded to L≥500 m. This observation, which focuses specifically on the informational behavior of the phenomenon, is in accordance with a recent analysis of the distribution of urban population, which exhibited multifractal behavior for scales over 800 m [36].

The analysis supported in Equations (9) and (10) was completed for q=[10,10] with Δq=1 to obtain the estimation of the multifractal spectra. It was observed that by making the time scale coarser, $D_{0}$ and $D_{1}$ grew in most cases. The growth of $D_{0}$ reveals that more criminal complaints were aggregated and, therefore, the occupation of the support from the observed phenomena became more noticeable. Besides having bigger fractal dimensions, $D_{0}$ in the weekly scale is an indicator of a geometry with a less-porous spatial structure.

The multifractal spectra spanned over an interesting set of local scales, indicating the strong mutlifractality characteristic of the objects in both temporal scales. The temporary aggregation allowed the levels of informational entropy to increase for the lowest spatial scales. However, information loss between spatial scales became more noticeable given the increase in $D_{1}$. Note also that the wide of spectra remained practically the same with the change of the temporal scale, corresponding to similar multifractal behaviors. Therefore, the perception of disorder remained similar despite the temporal aggregation of criminal complaints.

### 4.2. Cumulative Concavity Index

Results from the concavity test expressed in the CCI are presented in Table 2, according to the daily and weekly temporal scales and several $L_{min}$ for the computation of the dynamic multifractal spectrum f(α(n)). In the case of the daily scale, the test was exceeded on average for $L_{min}=1000$ m, whereas for smaller $L_{min}$ the dynamic multifractal spectra exhibited a significant degeneration. Only San Francisco and Philadelphia passed the test when $L_{min}=500$ m in the daily scale.

The CCI clearly improved for the weekly scale. On average, the concavity test became positive for $L_{min}\geq 500$ m. Most of the cases reported satisfactory concave indexes for $L_{min}=250$ m, as opposed to the daily case. These results are in accordance with the informational scaling shown in Figure 6, where the convenience of these spatio-temporal scales was noted for subreports with the largest number of criminal complaints.

### 4.3. MF $D_{1}$ Time Series

**Figure 8:**
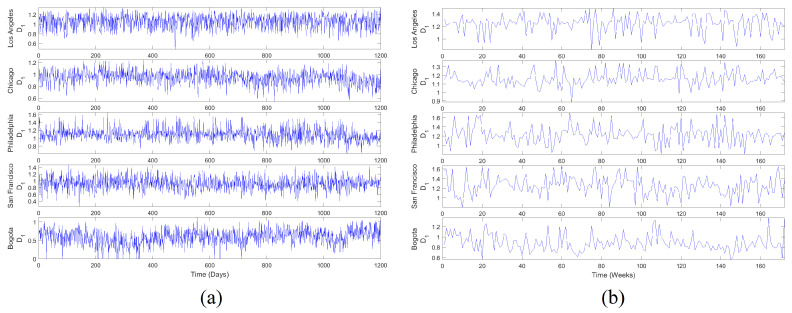
MF $D_{1}$ time series: (**a**) Daily scale, generated with $L_{min}=1000$ m; (**b**) Weekly scale, generated with $L_{min}=500$ m. MF $D_{1}$ series were generated from the dynamic multifractal spectra that achieved the best scores according to CCI. These series indirectly represent how spatial information production of urban crime fluctuates in time.

MF time series $D_{1}\left(n\right)$ were generated according to the MFA2OC method for two temporal scales, daily and weekly with $L_{min}=1000$ m and $L_{min}=500$ m, respectively. Results of this generation are presented in Figure 8. Note that these signals were characterized by interesting textures with fast and abundant fluctuations around several typical values. $D_{1}\left(n\right)$ signals in the weekly scale fluctuated more slowly with respect to the daily scale, which was a consequence of the spatial aggregation in wider time windows. The perception of disorder is evident and obvious patterns were not observed, evoking a preliminary hypothesis of randomness in the nature of these series and in the dynamics of spatial information.

### 4.4. Linear and Nonlinear Processing Results

**Figure 9:**
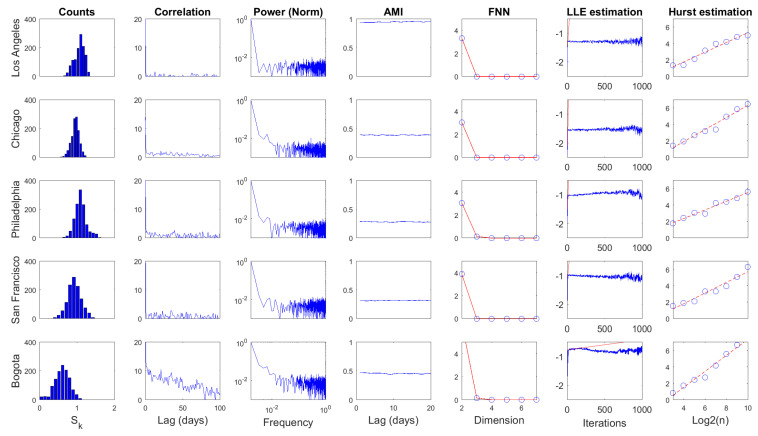
Processing results for $D_{1}\left(n\right)$, daily scale. The figure columns are organized from left to right as follows: histogram of the series, autocorrelation function, power spectrum, average mutual information, false nearest neighbors, largest Lyapunov exponent estimation, and Hurst exponent estimation.

**Figure 10:**
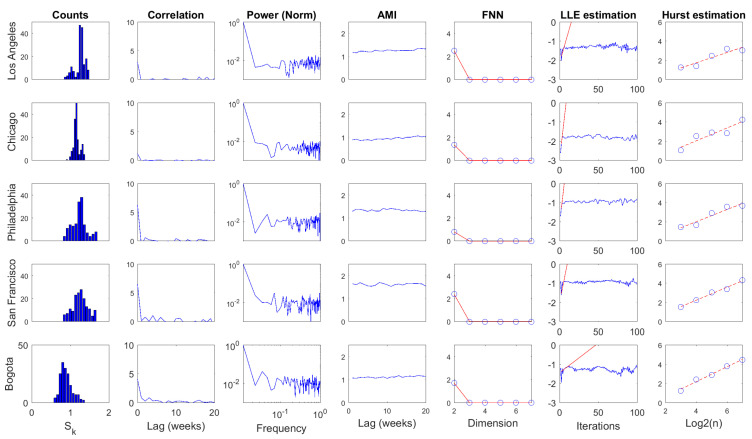
Processing results for $D_{1}\left(n\right)$, weekly scale. The figure is organized from left to right as follows: histogram of the series, autocorrelation function, power spectrum, average mutual information, false nearest neighbors, largest Lyapunov exponent estimation, and Hurst exponent estimation.

Several statistics were computed over $D_{1}$ time series for the daily and weekly scales, as shown in Figure 9 and Figure 10, respectively. The first column corresponds to the histogram of the series. Columns two and three present two statistics from linear processing: autocorrelation function and power spectrum. The following three columns depict nonlinear statistics: average mutual information (AMI), false nearest neighbors (FNN), and largest Lyapunov exponent (LLE)estimation. The last column depicts the estimation of the Hurst exponent. Some quantitative attributes regarding these results are presented in Table 3 and Table 4.

The basic statistical analysis of $D_{1}$ time series (i.e., histogram, mean, standard deviation std, and coefficient of variation CV) showed that information dynamics in urban crime for selected US cities fluctuated around similar levels with relatively small variations in both temporal scales. In contrast, information dynamics in the urban crime of Bogota evolved around a smaller level with significant fluctuations. Comparatively, the mean value of $D_{1}$ series increased from daily to weekly scale, generally preserving similar deviations, corresponding to the smallest CV in the latter scale.

The autocorrelation function revealed a fast decay of linear memory in daily $D_{1}$ series for US cases, whereas a slower decreasing was noted in the Bogota case. Linear memory in the weekly scale seemed to decrease similarly for all cases. The first minimum of the autocorrelation (CorrLag) function was located between two and five units in the daily scale. However, the span of the minimum was narrower in the weekly scale between only two and three units. In general terms, the temporal memory of $D_{1}$, understood in a linear fashion, did not extend over a long range for both scales.

The spectral centroids (Specent) of $D_{1}\left(n\right)$ were located in the low portion of the frequency spectrum for both temporal scales. This suggests that although high-frequency content was noticeable in these time series, their low-pass components were also significant in the dynamics of $D_{1}$. It can also be noted that power content decayed rapidly with frequency, which was more pronounced in US cases for the daily scale. In contrast, power decay would be similar for all cases in the weekly scale.

Regarding nonlinear statistics, it can be noted that significant AMI (Equation (18)) levels of $D_{1}$ series remained practically constant for wide temporal ranges. The first minimum of this function (AMILag) was between one and four time units in the daily scale, while in the weekly scale, the span of AMILag was narrower (i.e., between one and two time units). In both cases, the temporal decay of AMI seemed to be rapid. However, its small fluctuation indicates that these $D_{1}$ series exhibited strong nonlinear temporal memory. AMILag was used as *T* in Equation (17) to study $D_{1}\left(n\right)$ in the light of Taken’s theorem. The embedding dimension *d* was obtained from the FNN method as the value in which false neighbors dropped to zero EmbD. Results from FNN point out that these $D_{1}$ time series may be produced by deterministic dynamics that exist in low-dimensional spaces independent from the temporal scale.

The LLE estimation was carried out by means of Rosenstein’s method [55] with AMILag and EmbD as *T* and *d* in Equation (17), respectively. Divergence was noted in all cases for both temporal scales, which corresponds to the estimation of positive LLEs. This result suggests that the deterministic dynamics behind these $D_{1}$ time series are associated with low-dimensional chaotic attractors. The temporal scale in which these $D_{1}$ attractors were studied would not influence the perception of their chaotic motion. However, much faster divergences were estimated in US cases with respect to the Bogota case, which suggests the presence of attractors with stranger behavior.

Finally, the Hurst exponent estimation result was greater than 0.5 for all cases in both scales, indicating that these $D_{1}$ time series were irregular but persistent. Note that Hurst exponents increased significantly from daily to weekly scales, corresponding to series with more marked tendencies in the latter scale. However, note that a smaller set of scales was considered because of the reduced lengths.

## 5. Discussion

From the urban crime reports that were studied in this work, similar CCIs were computed for the weekly and daily generations of dynamic multifractal spectra. However, this sole criterion is not enough to make a decision about the convenience of an initial $L_{min}$ to study the multifractal characteristic of crime subreports given a temporal scale. It is necessary to go deeper into the complementary processing to study the dynamic behavior of $D_{1}$, which sheds light on the set of characteristic scales that should be considered when studying urban crime from its reports.

The evidence supported in the previous results suggests that the spatial information of selected urban crime cases, studied through MF $D_{1}$ time series, is generated by low-dimensional chaotic dynamics with strong nonlinear memory and persistent behavior in both daily and weekly scales. However, spatial scales of the studied phenomena started around $L_{min}=1000$ m (daily) and $L_{min}=500$ m (weekly), where the multifractal behavior can be detected and information scales with logL. In general terms, the dynamics of spatial information observed in these urban crime cases evolved around low levels of $D_{1}$ regarding the bidimensional support of crime reports.

The spatial information dynamics of urban crime exhibits a chaotic behavior in time. Although a deterministic production of information lies behind the core of urban crime, the low predictability of this phenomenon in space, time, and society is related to its chaotic informational dynamics. This consideration invites us to think that the complexity of crime emerges as a result of the interaction between the rational choice of agents and their interactions [4] with the information production of urban backcloths, in which those individuals are just a part of the whole [3].

Even though dynamic properties of $D_{1}\left(n\right)$ were studied in this work and helped us to understand scaling properties of urban crime from reported data, an ontological problem arises regarding these time series because they are not signals in a formal sense (i.e., a detectable physical quantity by which information is transmitted). A $D_{1}$ time series represents fluctuating information itself that emerges from a phenomenon. Accordingly, the notion of a physical quantity (i.e., state variable) associated to a nonlinear dynamical system is an open problem. If the case for the nature of $D_{1}\left(n\right)$ is solved as a dynamical variable related to others in Euclidean space, then the meaning of those variables would require a theoretical treatment beyond traditional information concepts.

One of the variables to which information production of crime may be related in dynamical terms is risk. There is evidence from the RTM practice that risk related to certain features of the urban backcloth is also a dynamic variable [70]. At the core, informational processes behind crime and risk may share common features or causal relations. This is an opportunity to consider measures of mutual information between risk and crime patterns as a quantitative tool to complement RTM methods. In this sense, multifractal analysis provides a conceptual framework to test the informational similarity between these patterns in multiple scales where information scaling of both phenomena is guaranteed. The spatial influence of risk was analyzed over a single scale that was selected from theory and empirical research [71], which may hinder the detection of patterns if distributions are not uninformative at that scale. Introducing the multifractal/informational approach in RTM may complement the way of experience and theory in finding suitable scales by looking at the data of the phenomenon.

The presence of temporal structure in the informational signals of crime dynamics also invites one to think about making inferences supported on cross-correlation measures with informational signals of risk computed from the MF-A2-OCD method. From the literature, it can be seen that RTM methods are supported on the ground of linear statistics when trying to find independent variables. It is known that nonlinear correlations can fool traditional statistics [30]. Hence, information-based measures like the AMI can be considered as detectors of nonlinear correlations between criminogenic variables.

$D_{1}\left(n\right)$ can be understood as a signal that represents the dynamics of urban crime in a surrogate fashion. Although these signals exhibited complex textures at daily and weekly scales, they evolved in deterministic chaotic motion with strong nonlinear memory. This quantitative result supports the idea at the heart of crime pattern theory about the non-randomness of crime. The evidence presented in this work indicates that the spatial information production of crime is not a stochastic temporal process. However, this does not mean that its dynamics is trivial. On the contrary, it is a challenge to model the dynamic equations that govern it. In addition, as information production in crime evolves chaotically, it is an indicator of non-stationary spatial patterns.

## 6. Conclusions

In this paper, we carried out a data-driven investigation of the information dynamics in reported urban crime. This dynamic was explored by means of a novel conjugation of multifractal analysis and some processing tools related to chaotic time series analysis. Our results suggest that information dynamics in crime evolve in a low-dimensional chaotic attractor. This can be observed in available crime reports in different spatio-temporal scales. However, certain scales are more favorable than others regarding the temporal properties of spatial information scaling, which is captured through the dynamic multifractal spectrum in its information dimension $D_{1}\left(n\right)$.

This work suggested the use of an information-based method to identify the set of spatio-temporal scales in which urban crime dynamics should be studied given a report of crime data. The key point of this method is to identify the scales in which the multifractal characteristic of urban crime report becomes evident. The identification is possible by looking at the integrity of the dynamic multifractal spectrum and the properties of $D_{1}\left(n\right)$ in terms of its temporal structure. Although the method was proposed specifically for studying crime, it could be used to study other kind of complex phenomena.

The findings presented in this paper support some theoretical perspectives that intend to explain urban crime as a phenomenon that emerges from complex urban systems. Information production can be considered as one of the elements that characterize the footprint of complexity in natural and socio-technical systems. However, the theoretical background that connects information production and nonlinear dynamics should be developed in an attempt to approach the complexity of this kind of system. Moreover, multifractal and nonlinear approaches combined through the MF-A2-OCD method can be considered as complementary tools to the practice of the risk terrain modeling of crime.

## Figures and Tables

**Table 1:** Some relevant features of cities and criminal reports. Four cities in North America (NA) and one city in South America (SA) are considered in this study.

Case	Area (km${}^{2}$)	Population (Billions USD)	Report Size (Complaints)	Mean Daily Complaints (Complaints per Day)
Los Angeles (NA)	50.7×70.8	12.15	860.45	121,974	99
Chicago (NA)	34.1×42	8.6	563.18	57,745	47
Philadelphia (NA)	26.7×31.9	5.44	346.45	91,806	75
San Francisco (NA)	13.5×13.9	3.36	331.02	19,683	16
Bogota (SA)	24.5×42.9	8.08	159.85	23,577	19

**Table 2:** Cumulative concavity index (CCI) results. CCI Was computed for dynamic multifractal spectra generated with three minimal spatial scales $L_{min}$ and two temporal scales: daily and weekly.

$L_{\min}$ (m)	CCI (Daily Scale)			CCI (Weekly Scale)		
	250	500	1000	250	500	1000
Los Angeles (NA)	0.7500	0.6564	0.9742	0.8039	0.9755	0.9804
Chicago (NA)	0.6404	0.6507	0.9692	0.8798	0.9663	0.9760
Philadelphia (NA)	0.2889	0.9602	0.9767	0.9808	0.9760	0.9760
San Francisco (NA)	0.6188	0.9605	0.9757	0.9709	0.9757	0.9806
Bogota (SA)	0.8382	0.8732	0.9244	0.9657	0.9771	0.9771
Average	0.6273	0.8202	0.9640	0.9202	0.9741	0.9780

**Table 3:** Quantitative attributes obtained from linear signal processing. Results show that information production in urban crime exhibited considerable fluctuation, short linear memory and wide frequency content in both temporal scales.

Statistic	Mean		Std		CV		CorrLag		Specent	
Scale (Time)	Daily	Weekly	Daily	Weekly	Daily	Weekly	Daily	Weekly	Daily	Weekly
**Los Angeles (NA)**	1.0578	1.2499	0.1367	0.1339	0.1292	0.1071	2	2	0.3342	0.2121
**Chicago (NA)**	0.9481	1.1616	0.1079	0.0838	0.1138	0.0722	4	2	0.2995	0.1606
**Philadelphia (NA)**	1.0936	1.2301	0.1536	0.1935	0.1405	0.1573	2	2	0.3328	0.2583
**San Francisco (NA)**	0.9252	1.2490	0.1839	0.1977	0.1988	0.1583	5	2	0.3774	0.2373
**Bogota (SA)**	0.5940	0.8870	0.1985	0.1556	0.3342	0.1755	2	3	0.3820	0.2356

**Table 4:** Quantitative attributes obtained from nonlinear signal processing. Results show that information production in urban crime evolved as low-dimensional chaotic attractors that exhibited strong nonlinear memory in both temporal scales.

Statistic	AMILag		EmbD		LLE		Hurst	
Scale (Time)	Daily	Weekly	Daily	Weekly	Daily	Weekly	Daily	Weekly
**Los Angeles (NA)**	1	2	3	3	76.1373	280.0472	0.5913	0.8043
**Chicago (NA)**	2	1	3	3	294.2945	698.2849	0.7356	0.8264
**Philadelphia (NA)**	2	1	4	3	156.3149	705.9980	0.5263	0.8143
**San Francisco (NA)**	3	2	3	3	53.3821	410.1712	0.6488	0.7790
**Bogota (SA)**	4	2	4	3	0.7062	61.7643	0.9718	0.8870
